# Improving Soybean Seed Sucrose Content using TILLING by Sequencing Analyses of The Soybean Sucrose Synthase Gene Family

**DOI:** 10.3389/fpls.2025.1606321

**Published:** 2025-06-25

**Authors:** Dounya Knizia, Erdem Anil, Yasser Salhi, Haiying Shi, Abdelhalim El Baze, My Abdelmajid Kassem, Naoufal Lakhssassi, Henry T. Nguyen, Khalid Meksem

**Affiliations:** ^1^ School of Agricultural Sciences, Southern Illinois University, Carbondale, IL, United States; ^2^ Division of Plant Science and Technology, University of Missouri, Columbia, MO, United States; ^3^ Plant Genomics and Biotechnology Laboratory, Department of Biological Sciences, Fayetteville State University, Fayetteville, NC, United States; ^4^ Department of Biological Sciences, School of Science, Hampton University, Hampton, VA, United States

**Keywords:** soybean, glycine max, sucrose synthase gene family, sucrose, raffinose, stachyose, EMS mutagenesis, TILLING

## Abstract

Soybean seed quality is influenced by its soluble sugar composition, with high sucrose content being desirable for nutritional and industrial applications. In contrast, excessive raffinose and stachyose levels are considered undesirable due to their adverse effects on gastrointestinal function in humans and monogastric animals. Therefore, developing soybean mutant lines with elevated sucrose content and optimal raffinose and stachyose content is desirable. In this study, we characterized twelve sucrose synthase genes through a comprehensive phylogenetic tree analysis, synteny analysis, gene structure evaluation, and variations in conserved domains. Additionally, we conducted a TILLING by Sequencing approach to identify EMS mutations in the characterized Sucrose synthase genes. Numerous mutations have been identified in soybean sucrose synthases that resulted in high sucrose content, including the sucrose synthases mutants SL446 (R582W) and F1115 (G249E) on *Glyma.02G240400* with a sucrose content of 9.5% and 9.1%, respectively. The obtained soybean mutants with enhanced sugar content can be useful in breeding programs to improve soybean nutritional quality without potential developmental trade-offs.

## 1 Introduction

Soybean is one of the most important crops worldwide providing many nutrients, including protein, oil, and carbohydrates for animal and human consumption. Soybean seeds contain approximately 35% carbohydrates, including soluble sugar and fibers (Middelbos and Fahey, 2008). The sucrose, raffinose, and stachyose represent 5-7%, 1%, and 4-6% of total carbohydrates, respectively of soybean seed dry weights (Skoneczka et al., 2009). The soluble sugar composition determines the quality of soybean and its nutritional value. The sucrose is desirable because it gives the soybean seeds sweetness and is a major source of energy for the fermentation of soy-based products such as natto and tofu (Hou et al., 2009). Additionally, high levels of raffinose and stachyose are considered antinutritional because humans and monogastric animals cannot digest them due to the lack of α-galactosidases. This can lead to reduced gastrointestinal performance, flatulence, or diarrhea (Arunraj et al., 2020; Zhang et al., 2019).

Sucrose is a disaccharide composed of two monosaccharides: glucose and fructose. It is the final product of photosynthesis, and the primary sugar translocated in the plant phloem from the source leaves to sink tissues of growth, development, and energy storage (Koch, 2004; Salerno and Curatti, 2003). Sucrose biosynthesis occurs only in photosynthetic tissues or in germinating seeds. Generally, sucrose is stored in vacuoles and used whenever the cell requires more energy. Sucrose catabolism is a vital metabolic process that regulates carbon flux and initiates sugar signaling. This process is primarily driven by two enzymes: sucrose synthase and invertase. Sucrose synthase catalyzes a reversible reaction, utilizing UDP to produce UDP-glucose and fructose, and can also contribute to sucrose synthesis. In contrast, invertase functions as a hydrolytic enzyme, irreversibly breaking down sucrose into glucose and fructose. Sucrose synthase is a member of the glycosyl transferase enzyme subfamily 4 (GT-4) that is crucial for a plant’s sugar metabolism. The sucrose synthase enzyme catalyzes a reversible reaction of sucrose with UDP or ADP to fructose and diphosphate glucose (UDP-G or ADP-G). The resulting products are useful for the cell’s metabolic pathways, including energy production and synthesis of other carbohydrate molecules. This enzyme is usually located in the cell’s cytosol or adjacent to the plasma membrane (Stein and Granot, 2019). The sucrose synthase protein is a tetramer composed of monomeric units with a molecular weight of about 90 kDa and a sequence of approximately 800 amino acids long, with small variations between different isozymes (Stein and Granot, 2019). Each monomer of the sucrose synthase protein contains four distinct domains including a cellular targeting domain (CTD, residues 11–127), an ENOD40 peptide-binding domain (EPBD, residues 157–276), and two domains that comprise the glycosyltransferase (GT-B) involved in the enzyme’s glycosyltransferase activity. The two glycosyltransferase (GT-B) domains are referred to as N- and C-terminal domains (GT-BN and GT-BC) (Lairson et al., 2008). The C-terminal glycosyltransferase domain extends from residues 277 to 526, while the N-terminal glycosyltransferase domain extends from residues 527 to 754 (Zheng et al., 2011). The substrates (UDP-Glucose or UDP and fructose) bind to the sucrose synthase protein in the clef between the GT-BN and GT-BC (Zheng et al., 2011). Sucrose synthase enzyme possesses two serine phosphorylation sites including the first site, which is located between locations 11 to 15 and plays a role in membrane association, while the second one is involved in protein degradation and is located between residues Glu14 and Met193 (Hardin et al., 2003; Zhang et al., 1999). Additionally, the Cys264 residue has been identified as a binding site located in the ENOD40 peptide A, which activates the cleavage activity of the sucrose synthase site (Röhrig et al., 2004). Interestingly, the sucrose synthase enzyme functions using a ‘hinge-latch’ mechanism that transitions between open and closed forms, allowing substrate binding and catalysis (Wu et al., 2015). In plants, the sucrose synthase enzyme uses preferably UDP-glucose compared to the prokaryotes’ sucrose synthase which prefers to use ADP-glucose as a substrate (Wu et al., 2015).

TILLING (Targeting Induced Local Lesions IN Genomes) by target sequencing is a reverse genetic approach that combines induced mutations from a chemical mutagenized population with high-throughput mutation screening methods to characterize the genes and better understand their functions. The chemical mutagenesis using ethyl methane sulfonate (EMS) is considered the most common chemical mutagenesis used to create random point mutations across the plant genome (Koornneef et al., 1982). The TILLING technology has been used to investigate the gene function related to the most economically important traits in soybean, including seed composition (Dierking and Bilyeu, 2009; Lakhssassi et al., 2024, 2021a, 2021b, 2017, 2020; Zhou et al., 2019, 2021) and disease resistance (Dierking and Bilyeu, 2009; Lakhssassi et al., 2022).In the current study, we characterized the soybean sucrose synthase candidate genes identified in (Knizia et al., 2023). This characterization was performed through a comprehensive phylogenetic tree analysis, synteny analysis, gene structure, and conserved domain variations. We also used TILLING by Sequencing to identify EMS mutations in the sucrose synthase genes characterized genes.

## 2 Materials and methods

### 2.1 Identification of sucrose synthases from soybean and other plant species

The sequences of the soybean sugar synthase genes were obtained from the soybean reference genome (Glycine max, Wm82.a2.v1) (Goodstein et al., 2012; Schmutz et al., 2010). The sucrose synthase protein sequences from other species, including *Arabidopsis thaliana*, *Phaseolus vulgaris*, *Medicago truncatula*, *Zea mays*, *Triticum aestivum*, *Beta vulgaris*, *Selaginella moellendorffii*, *Physcomitrium patens* and *Sorghum bicolor*, were identified by using the enzyme name as a query in search of each species in the available data at Phytozome database (Goodstein et al., 2012). A total of 50 gene sequences for the sucrose synthase enzyme were used in this study.

### 2.2 Phylogenetic analysis

MUltiple Sequence Comparison by Log- Expectation (MUSCLE) was used to align the protein sequences of 9 plant species. The resulting file was used to construct an unrooted phylogenetic tree using the maximum likelihood (ML) method in MEGA 11 using the Jones-Taylor-Thornton Gamma Distributed (JTT+G) model. The tree topology robustness was checked with a bootstrap analysis of 1000 replicates (Hall, 2013; Tamura et al., 2021).

### 2.3 Gene structure and expression profiling

The soybean sucrose synthase genomic and coding sequences were obtained from Phytozome v13 (Goodstein et al., 2012) and aligned to create the gene exon-intron structure diagram using the Gene Structure Display Server to create the gene exon-intron structure diagram (Hu et al., 2015).

The RNA seq data from different plant tissues including leaves, flowers, pods, pod shells, seeds, roots, and nodules was retrieved from the publicly available data from SoyBase (Brown et al., 2021). The heatmap was constructed using the Heatmapper (Babicki et al., 2016).

### 2.4 Chromosomal distribution and syntenic analysis

The Persephone software was used to conduct syntenic analysis on the soybean genome. The map of the Williams 82 genome annotation a2.v1 (W82.a2.v1) was used to identify the duplicated regions in soybean chromosomes by selecting the chromosome carrying our gene of interest and looking for the synteny. The synteny refers to the conservation of blocks of order within two sets of chromosomes, between the selected chromosome and the rest of the soybean chromosomes. Once the duplicated regions are identified, we zoomed into the precise location of our gene of interest to locate the corresponding duplicated gene in the other chromosome. Information about the homologous genes and the duplicated regions was collected, and the chromosome maps were constructed. The soybean chromosomes and the sucrose synthase gene locations were illustrated according to the soybean genome annotation a2.v1 on SoyBase (Brown et al., 2021).

### 2.5 TILLING by Sequencing+

The sucrose synthase genes used in this study were identified in (Knizia et al., 2023). The genes corresponding glyma numbers and accession numbers were provided in **Table S1**. The details of the TILLING by Sequencing+ have been described before in several publications from our lab (Lakhssassi et al., 2021a; Zhou et al., 2021). Below is a summary of the steps that were used:

#### 2.5.1 EMS mutagenesis and mutant population development

A mutagenesis test was conducted using ten different EMS concentrations, ranging from 0% to 1.0% (v/v) to determine the optimal EMS concentration to develop a large population of mutants. The specific concentrations tested were 0%, 0.2%, 0.3%, 0.4%, 0.5%, 0.6%, 0.65%, 0.7%, 0.8%, and 1.0%. A set of one hundred soybean (Glycine max) seeds from the wild-type cultivars ‘Forrest’, Saluki’, PI407729, PI88788 and PI567516C, were soaked inside a fume hood in ten different concentrations of EMS in a volume of 100 ml at room temperature for 16 hours. Then, the treated seeds were washed 3 times using 300 ml of water per wash to remove excess EMS from the seeds. The water used to rinse the treated seeds was neutralized with a 10% (w/v) sodium thiosulfate solution. The treated seeds were planted in 48-cell trays filled with ProMix BX (Premier Tech., Rivière-du-Loup, Québec, Canada) and grown in the greenhouse at the Horticulture Research Center (HRC) at Southern Illinois University Carbondale. The plants were grown at 28-30°C under a 16 h light/8 h dark photoperiod. The germination rate of each treatment was recorded after 10 days, and the treatment showing an LD50, compared to the wild type, was used to mutagenize the large mutant population (Meksem et al., 2008).

Initially, a total of 4000 seeds from each cultivar were treated with 0.6% (v/v) EMS solution for 16 hours at room temperature inside the fume hood. The treated seeds were washed thoroughly three times with water the following day. Afterward, the mutagenized seeds (M1) were sown in ProMix BX soil and grown in the greenhouse at 28-30°C under a photoperiod of 16 h light/8 h dark. After reaching the V2-V3 vegetative growth stages (having two to three sets of unfolded trifoliolate leaves), the seedlings were transplanted to the field at the Horticulture Research Center (HRC) at Southern Illinois University Carbondale. The M1 plants were drip-irrigated and were grown in the field until they reached full maturity. Then, the M2 seeds, produced by M1 plants through self-pollination, were harvested, threshed, packaged, and stored in the seed lab at -20°C. In the following planting season, the M2 seeds were planted in the greenhouse at HRC using the single-seed descendent method. The seedlings were grown at 28-30°C under a photoperiod of 16 h light/8 h dark until they reached the V2-V3 vegetative growth stages, then young leaf tissues from each M2 plant were collected and stored at -80°C for DNA extraction. Afterward, the plants were transplanted into the field at HRC and grown there until they reached full maturity. The M3 seeds were next harvested, threshed, and stored for phenotypic analysis.

#### 2.5.2 DNA extraction and quantification

50–100 mg leaves tissue samples collected from each M2 plant of the EMS population were disrupted with tungsten carbide beads in a 96-well plate using the TissueLyser System (QIAGEN, Valencia, CA, USA). The DNeasy 96 Plant Kit (QIAGEN, Valencia, CA, USA) was used to perform DNA extraction. DNA concentration was estimated with the spectrophotometer Synergy 2 Multi-Mode Microplate Reader (BioTek Instruments Inc., Winooski, VT, USA) and then normalized at 100 ng/µL. The extracted DNA samples were stored at -20°C.

#### 2.5.3 Library preparation, probe design, and sequencing

A bidimensional-arraying strategy was performed to pool the genomic DNA from 76 96-well plates to enhance the screening throughput. DNA arrayed from the same row of each four consecutive plates was combined in one column of P1 and P2 for the horizontal pools. Each well of the horizontal pools contains 48 samples from the source DNA plates. Moreover, DNA from the same column of three non-consecutive plates, with four plates in between, was pooled together for the vertical pools. For instance, plate “1” is followed by plate “5” (Pn, Pn+4). This pooling was done in one row of plates P3, P4, P5, and P6. Each well in the vertical pool contains 24 DNA samples.

The platform capture-seq at Rapid Genomics LLC (Gainesville, FL, USA) carried out the probe design and synthesis to cover the exons of each gene: the preparation of the DNA libraries, the capture enrichment (by magnetic beads), and the next-generation sequencing (Illumina HiSeqX 2x150 bp).

#### 2.5.4 Single nucleotide polymorphism calling and bioinformatics pipeline

The obtained raw data from the sequencing were in a FASTQ format that was tested for quality using FastQC v0.11.9 (Andrews, 2010), followed by trimming, filtering, and discarding the low-quality reads using Trimmomatic (Bolger et al., 2014). The cleaned FASTQ reads were aligned to the Williams 82 (Wm82.a2.v1) reference genome using BWA (Li and Durbin, 2009). The obtained Binary Alignment Map (BAM) files were filtered and sorted using the SAM tools v1.10 (Li and Durbin, 2009), and the output was sorted Sequence Alignment Map (SAM) file format that was used as an input for variant calling using CRISP v1.18.0 (Huang et al., 2015). The generated VCF files were filtered by VCFtools (AlbersCornelis et al., 2011) to retain the true induced mutations and then visualize using IGV for demultiplexing (Robinson et al., 2011). The VCF files have a description of each mutation including the type of mutation, position, allele frequency (AF), quality score (QUAL), and alternative allele counts (AC). The reference genome WI82.a2.v1 (Schmutz et al., 2010) was utilized for SNP calling and predicting mutation effects. To confirm the mutations, the isolated TILLING mutants were target-sequenced using Sanger sequencing.

#### 2.5.5 Mutation validation

Sanger sequencing was used to confirm the identified mutations in soybean sucrose synthase genes. Twelve seeds from each identified mutant were planted in the greenhouse at the HRC, and young leaves were collected from each plant for DNA extraction. DNA was extracted from each plant tissue using the CTAB method. Meanwhile, specific PCR primers were designed to amplify the fragments covering the exons of sucrose synthase genes using Primer3 (Koressaar and Remm, 2007) (APPENDIX A). The PCR program was set up with 40 cycles of amplification at 94°C for 30s, 58°C for 30s, and 72°C for 1 min. The resulting PCR products were purified by either enzymatic clean-up or the specific bands were retrieved from the agarose gels after electrophoresis using the QIAquick○R PCR Purification Kit (QIAGEN, Valencia, CA, USA), and the QIAquick○R Gel Extraction Kit (QIAGEN, Valencia, CA, USA) The purified samples were sequenced by GENEWIZ (https://www.genewiz.com/). The mutations were identified by alignment of the mutant sequence to the reference sequence (wild type) using Unipro UGENE (Okonechnikov et al., 2012).

### 2.6 Seed sugars (sucrose, raffinose, and stachyose) analysis

The HPLC method used in this study was a modification of the original method published in 2015 (Valliyodan et al., 2015). Mature seeds from the wild types and the mutagenized mutants were analyzed for sugar contents including sucrose, raffinose, and stachyose using an Agilent HPLC-ELSD 1200 series equipped with an evaporative light scattering detector (ELSD) and a Waters xBridge BEH amide column (5µ, 250 × 4.6mm) with VanGuard Cartridge (3.9 X 5 mm). About 5 g of soybean seeds were finely ground into a powder using Thomas Wiley Mini-Mill (Arthur Thomas Co., Philadelphia, PA, USA) with a 20-mesh screen. The powder was subsequently lyophilized for two days in a Labconco freeze-dry system (Labconco, USA). Approximately 90.20 ± 0.20 mg of seed powder was mixed with 900 μL HPLC-grade water in a 2 mL centrifuge vial. Afterward, the vials were incubated at 55°C with agitation at 200 rpm for 1 hour, followed by a 30-second high-speed vortex. 900 μL HPLC grade acetonitrile was added to each vial after cooling at room temperature for 45 minutes. The suspension was centrifuged for 30 minutes at 14,000 × g. The supernatant was diluted fivefold with 65% HPLC-grade acetonitrile before undergoing HPLC analysis. Sugar standards of sucrose, raffinose, and stachyose were prepared in HPLC-grade water at final concentrations of 50, 100, 300, 500, and 1000 μg/mL. The HPLC instrumental method used two mobile phases: mobile phase A (pure water) and mobile phase B (acetonitrile). A gradient program of phases A and B was applied to separate the three sugars within a 20-minute timeframe. The column temperature was maintained at 35°C, while the detector temperature was set to 55°C. The detector pressure was set at 3.4 bar, and ultra-purity-grade nitrogen (grade 5.0) was used as the carrier gas. The sample injection volume was 5µL, and the flow rate was set to 1.2 ml/min.

### 2.7 Conserved domains analysis and homology modeling of sucrose synthase genes

Multiple sequence alignment between sucrose synthase gene family of *Glycine max*, *Arabidopsis thaliana*, *Phaseolus vulgaris*, *Medicago truncatula*, *Zea mays*, *Triticum aestivum*, *Beta vulgaris*, *Selaginella moellendorffii*, *Physcomitrium patens* and *Sorghum bicolor* was performed using MUSCLE alignment, Catalytic residues in conserved motifs of soybean sucrose synthase were identified from NCBI Conserved Domain Database (CDD) (https://www.ncbi.nlm.nih.gov/cdd).

Homology modeling of putative sucrose synthase protein structures was conducted with Deepview (Guex et al., 2009) and Swiss Model Workspace software (Waterhouse et al., 2018) using the protein sequence from “Williams 82” and an available crystal structure as a template; PDB accession 3s27.1 for sucrose synthase. The structure visualization and mutation mapping were performed using the UCSF Chimera package (Pettersen et al., 2004). The conserved domain for each gene family was visualized using InterPro (https://www.ebi.ac.uk/interpro/) and Jalview multiple alignment editor program (https://www.jalview.org/).

### 2.8 The assessment of the agronomic performance of the mutants

20 to 30 seeds from sucrose synthase mutants identified through TbyS and 20 seeds from the wild types of Forrest and Saluki were planted at the HRC at Southern Illinois University Carbondale to evaluate the impact of the confirmed mutations on the agronomical performance of the plants. The plants were sown in ProMix BX soil and grown in the greenhouse at 28-30°C under a 16 h light/8 h dark photoperiod. After reaching the V2-V3 stage, the seedlings were transplanted to the Horticulture Research Center (HRC) field at Southern Illinois University Carbondale. Drip irrigation was used to irrigate the plants. When the plants reached R4, measurements of the plant’s height were taken and the number of pods was counted, and three pictures were taken for each plant to show its state, the first one for the whole plant, another one for the leaves, and the last one for the pods. The statistical significance between the number of pods and the height of each mutant compared to the wild type was performed using the JMP Pro 16 software.

## 3 Results

### 3.1 Soybean sucrose synthase gene family characteristics

In total, 12 gene members belonging to the sucrose synthase gene family in soybean were previously identified (Knizia et al., 2023). The genomic sequence analysis of this gene family has shown that the number of exons of these genes varies between 12 and 15 (**Table 1**).

**Table 1:** The list of soybean sucrose synthase genes with their corresponding gene ID, nucleotide sequence characteristics, and protein sequence properties.

Gene ID	Gene length (bp)	CDS (bp)	Exons	Protein sequence (aa)	pI	Mol.Wt. (kDa)
Glyma.02G240400	5600	2523	14	841	6.81	95.33
Glyma.03G216300	6241	2439	15	813	5.76	92.31
Glyma.09G073600	6397	2433	12	811	6.02	93.66
Glyma.09G167000	5901	2766	15	922	6.91	105.38
Glyma.13G114000	5528	2418	12	806	6.04	92.24
Glyma.14G209900	5789	2523	14	841	6.01	86.86
Glyma.15G182600	6296	2421	13	807	5.87	92.72
Glyma.15G151000	10938	2409	15	803	5.84	91.57
Glyma.16G217200	5611	2763	15	921	6.66	103.91
Glyma.17G045800	5574	2418	12	806	6.34	96.1
Glyma.19G212800	6134	2439	15	813	5.95	92.26
Glyma.11G212700	5184	2538	15	846	6.53	95.77

The coding DNA sequences (CDS) length of the sucrose synthase genes varies between 2409 and 2766 with an average of 2507.5. Furthermore, the size of the sucrose synthase protein ranges from 803 to 922 amino acids with a molecular weight that varies between 91.57 and 105.38 kDa (**Table 1**).

Interestingly, some pairs of genes have similar genomic sequence length, number of exons, protein size, and CDS length including, *Glyma.13G114000/Glyma.17G045800, Glyma.03G216300/Glyma.19G212800, Glyma.02G240400/Glyma.14G209900*, and *Glyma.16G217200/Glyma.09G167000* for sucrose synthase (**Table 1**).

### 3.2 Phylogenetic analysis of plant sucrose synthase gene families

A BLAST search using soybean protein sequence was conducted to identify protein sequences of sucrose synthase genes from two legume species, *Phaseolus vulgaris* and *Medicago truncatula*; two dicot species, *Arabidopsis thaliana* and *Beta vulgaris*; three monocot species *Zea mays*, *Triticum aestivum*, and *Sorghum bicolor*; a Lycophytes species *Selaginella moellendorffii*; and a Bryophyta specie *Physcomitrium patens*.

To understand the evolutionary modules and their relationship to functional ones, we constructed a maximum likelihood tree using the protein sequences of 50 sucrose synthase genes, based on 1000-replicate bootstrap values.

As expected and reported in previous studies (Xu et al., 2019; Zhu et al., 2017), the phylogenetic tree classified the sucrose synthase genes into three groups I, II, and III.

**Figure 1:**
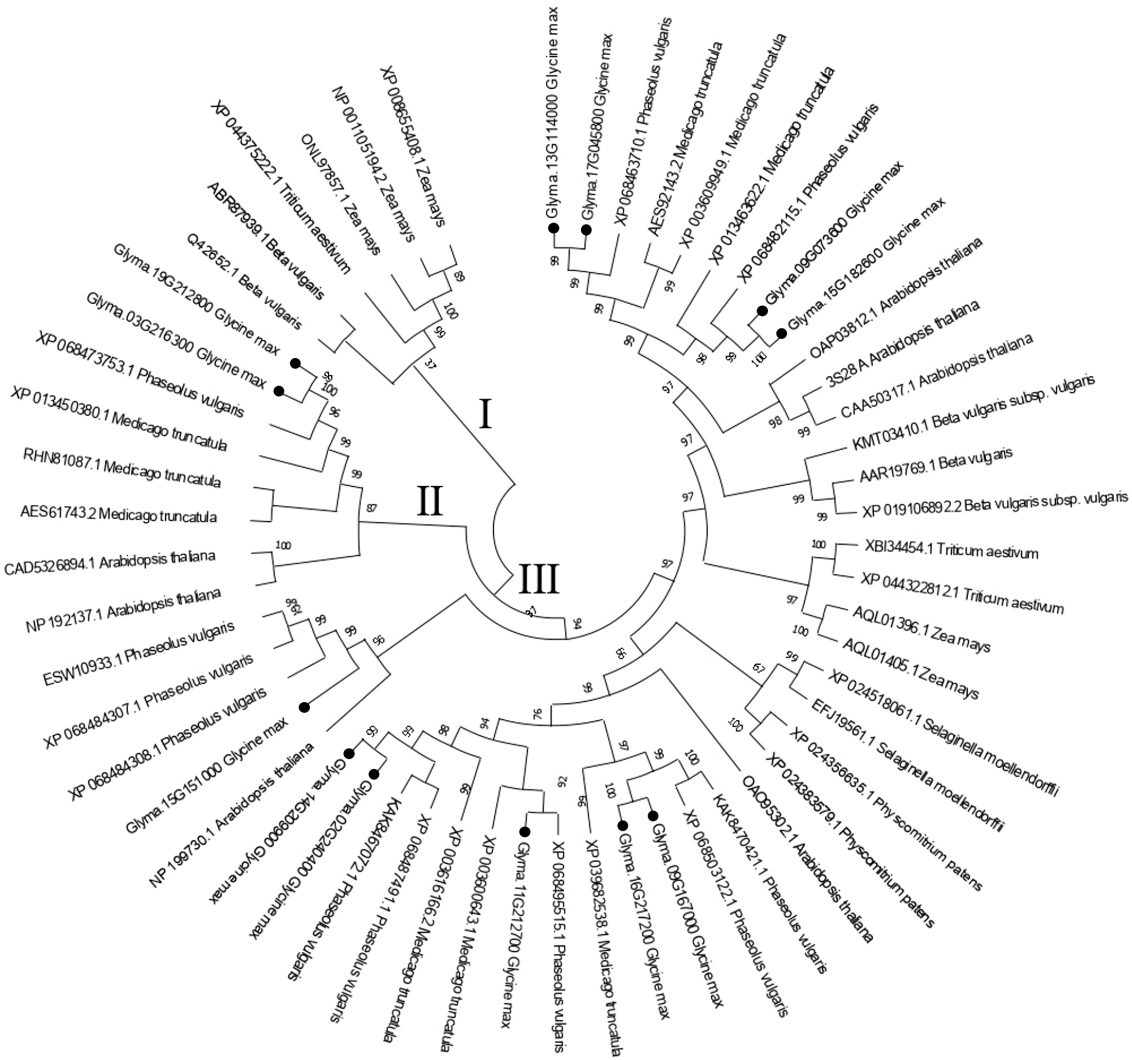
Phylogenetic gene tree for sucrose synthase gene family from nine plant species including Arabidopsis thaliana, Phaseolus vulgaris, Medicago truncatula, Zea mays, Triticum aestivum, Beta vulgaris, Selaginella moellendorffii, Physcomitrium patens and Sorghum bicolor. The protein sequences were subjected to a MUSCLE multiple alignment and a phylogenetic gene tree was constructed by the maximum likelihood (ML) method using Mega 11.

In group I, the genes are organized into two subgroups, one for the monocots and the other for the dicots. The soybean sucrose synthase genes *Glyma.13G114000* and *Glyma.17G045800* form a subclade, in the subgroup containing the dicots. Likewise, the *Glyma.09G073600* and *Glyma.15G182600* form another subclade in the same subgroup. Remarkably, Phaseolus vulgaris has genes that cluster with almost every soybean gene, indicating the presence of a common ancestor (**Figure 1**). Group II contains *Glyma.09G167000* and *Glyma.16G217200* clustered in the same subclade, and *Glyma.02G240400* and *Glyma.14G209900* were clustered in another subclade. While *Glyma.15G151000* forms a subclade by itself and *Glyma.11G212700* forms a subclade with a *Phaseolus vulgaris* sucrose synthase gene (**Figure 1**). In Group III, *Glyma.03G216300* and *Glyma.19G212800* are clustered into the same subclade (**Figure 1**).

Interestingly, the three groups contain monocots and dicots suggesting that most gene duplication events that gave rise to sucrose synthase gene groups happened before the monocot/dicot divergence (**Figure 1**).

### 3.3 Gene structure, expression profiling among sucrose synthase genes

**Figure 2:**
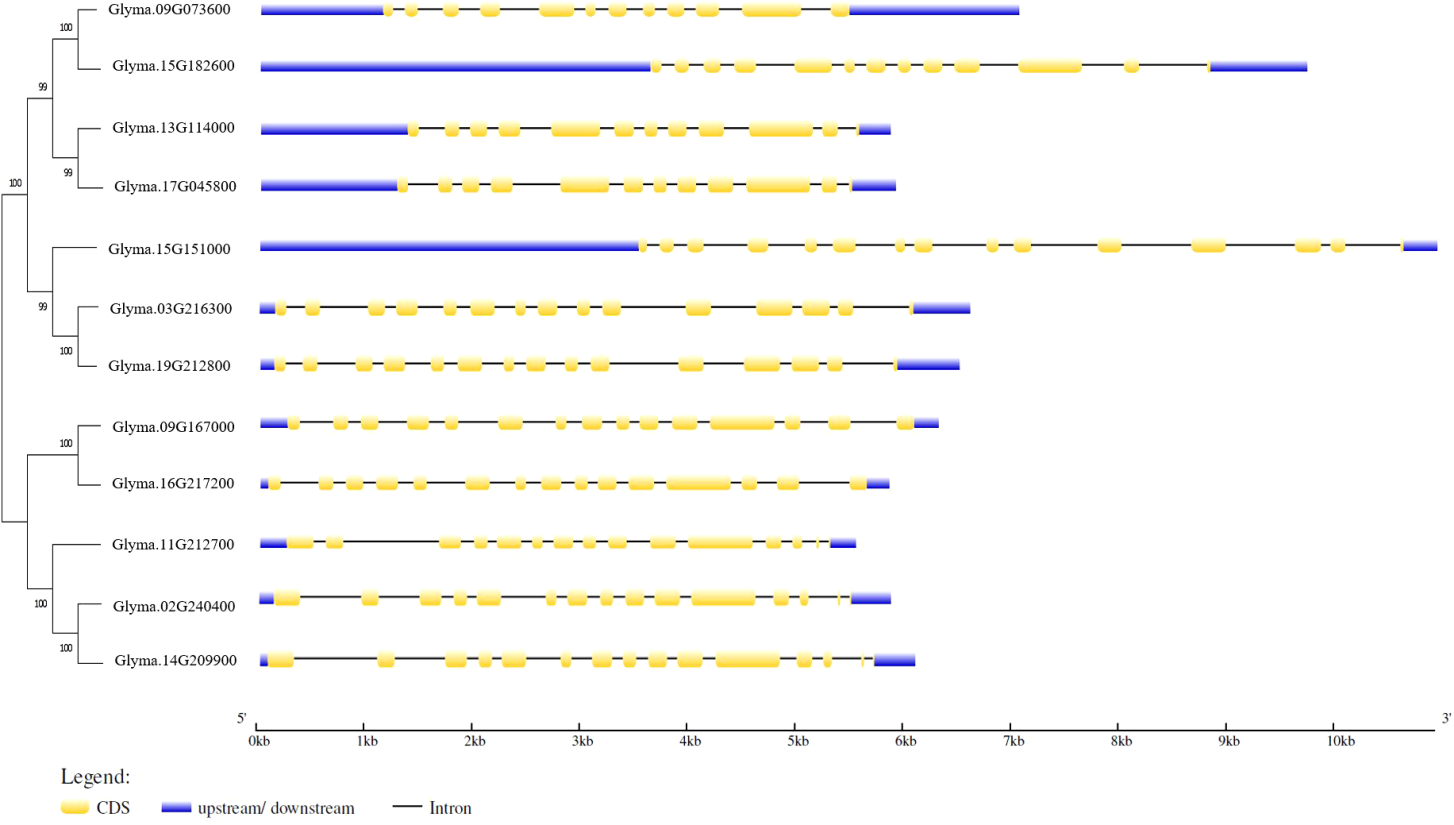
Phylogenetic relationships and gene structures of soybean sucrose synthase. The protein sequences of each gene family were aligned using MUSCLE and the phylogenetic tree was constructed using MEGA 11. The structures of 12 soybean sucrose synthase genes were illustrated with yellow boxes representing exons (coding DNA sequence, CDS), black lines illustrating introns, and blue boxes indicating 5’-UTR and 3’-UTR regions. The size of gene structures can be measured by the base pair (bp) scale at the bottom. The gene structure was drawn using the Gene Structure Display Server (Hu et al., 2015).

The soybean sucrose synthase gene family consists of 12 members, which is twice the amount found in Arabidopsis. The average genomic sequence length of this gene family is 6266 bp, *Glyma.15G151000* has the longest sequence due to its extended 5’UTR region (**Table 1**). The gene structure of the soybean sucrose synthase genes is conserved for only six gene members including *Glyma.15G151000, Glyma.03G216300, Glyma.19G212800, Glyma.11G212700, Glyma.16G217200*, and *Glyma.09G167000* that have 15 exons. Whereas *Glyma.13G114000, Glyma.17G045800, Glyma.09G073600* have 12 exons, *Glyma.02G240400* and *Glyma.14G209900* have 14 exons, and *Glyma.15G182600* has 13 exons (**Table 1** and **Figure 2**).

**Figure 3:**
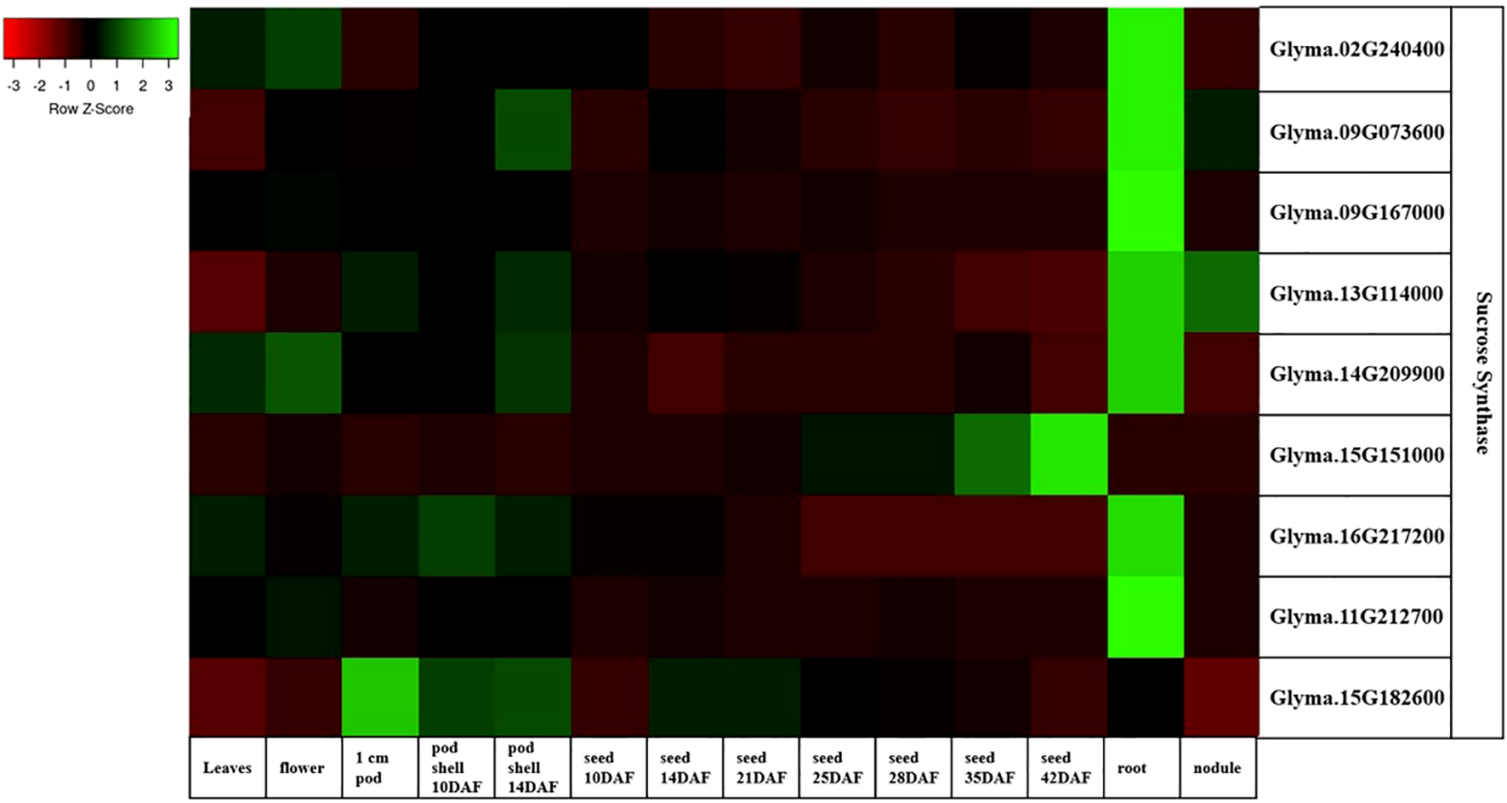
Expression heatMap of the soybean sucrose synthase genes in Williams 82 (RPKM) retrieved from publicly available RNA-seq data from the soybase database (Brown et al., 2021). The color key represents the relative transcript abundance from low (red) to high (green). RNA-seq data is not available at Soybase for *Glyma.17G045800, Glyma.03G216300*, and *Glyma.19G212800*.

To gain insight into the function of sucrose synthase genes in soybean seeds, RNA-Seq analysis was conducted to examine the expression profiles of these genes. The results have shown that the sucrose synthase genes: *Glyma.09G073600*, *Glyma.13G114000*, and *Glyma.15G182600* showed relatively higher expression profiles in all the examined tissues compared to the rest of the genes. Meanwhile, the *Glyma.15G151000* gene is highly expressed in the seeds (**Figure 3**).

In most tissues, the expression patterns of the sucrose synthase genes *Glyma.16G217200* and *Glyma.11G212700* are recorded as 0 in most of the tissues.

### 3.4 Chromosomal distribution and gene duplication

A syntenic analysis was conducted on the soybean genome. Duplicated chromosomal segments containing sucrose synthase genes were investigated to test the impact of the soybean gene duplication events on the sucrose synthase genes.

**Figure 4:**
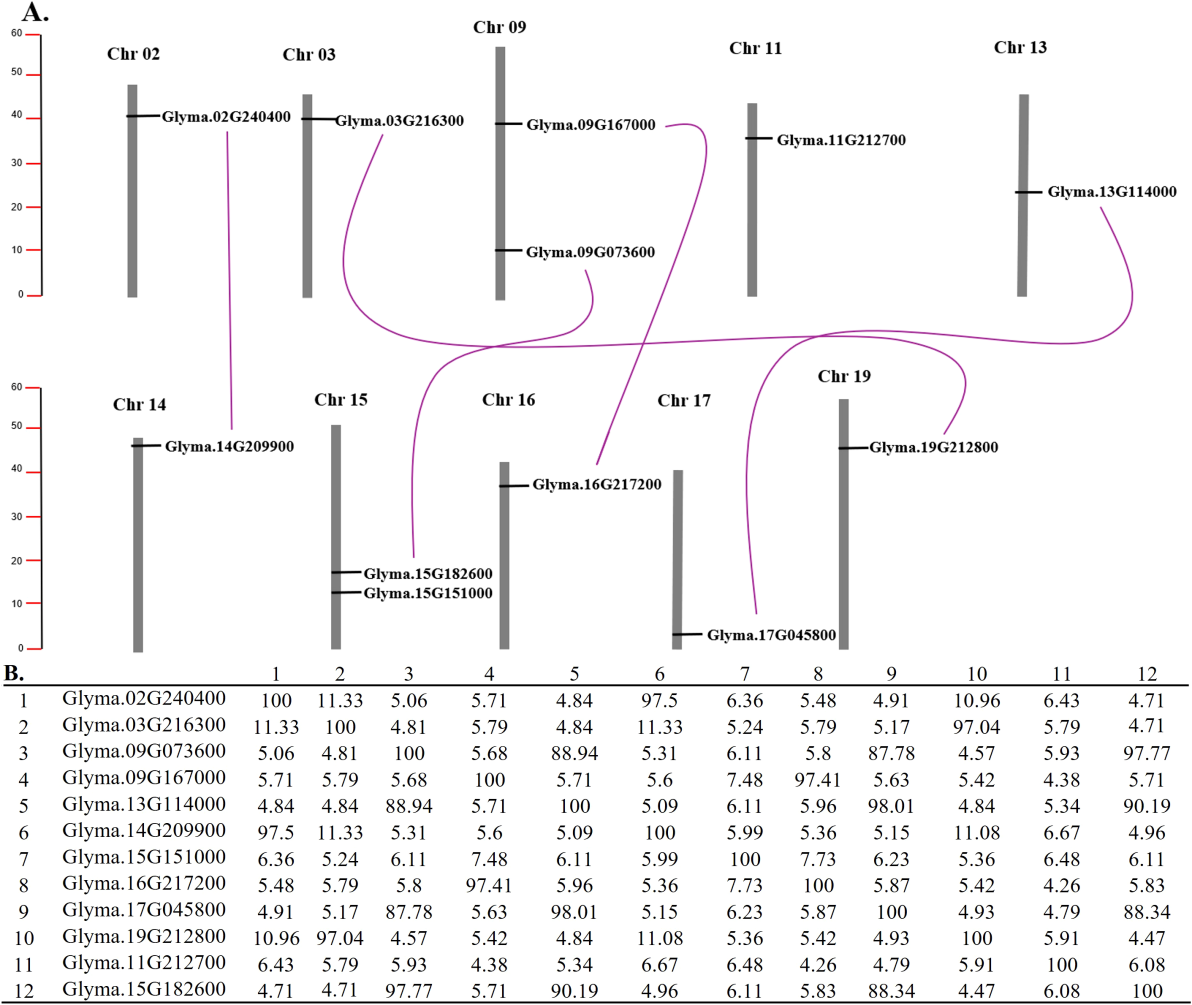
**(A)** Chromosomal locations and duplications of 12 soybean sucrose synthase. The chromosome size and the gene locations were drawn based on soybean genome annotation a2.v1 on SoyBase (Brown et al., 2021). The scale is on the left and it’s in megabase (Mb). Each duplicated pairs of sucrose synthase genes are linked by a purple line, respectively. **(B)** Percent Identity Matrix of the Sucrose Synthase Genes, created by Clustal2.1 using the multiple sequence alignment of the soybean sucrose synthase protein sequences performed at Mview (Multiple Sequence Alignment (MSA)) at EMBL-EBI (https://www.ebi.ac.uk/).

Twelve sucrose synthase genes are distributed on 9 different chromosomes in the soybean genome. Chromosomes 9 and 15 contain two genes each, while chromosomes 2, 3, 11, 13, 14, 17, and 20 have one gene each (**Figure 4**).

The results of the syntenic analysis performed on the soybean genome have shown the presence of five segmental duplications in 8 chromosomes that contain sucrose synthase genes. The first one is between chromosome 2 and chromosome 14. These regions contain the *Glyma.02G240400* and *Glyma.14G209900* sucrose synthase genes with 97.2% similarity (**Figure 4B**). The second duplication is between chromosome 3 and chromosome 19. *Glyma.03G216300* and *Glyma.19G212800* are involved in this duplication. These two genes have 97% similarity (**Figures 4A, B**). The third duplication is between chromosomes 9 and 16 involving *Glyma.09G167000* and *Glyma.16G217200* which has 91.41% similarity (**Figures 4A, B**). Another segmental duplication is found between chromosomes 9 and 15 involving *Glyma.09G073600* and *Glyma.15G182600* which shows 97.77% similarity (**Figure 4A, B**). The last segmental duplication is found between chromosome 13 and chromosome 17. *Glyma.13G114000* and *Glyma.17G045800* are also part of this duplication. These two genes show 98% similarity (**Figure 4B**). The results are consistent with phylogenetic analysis and gene structure and sequence analysis.

No tandem duplication events were detected in the soybean sucrose synthase genes.

### 3.5 Conserved domains of sucrose synthase enzymes

The sucrose synthase enzyme contains four domains: CTD, EPBD, GT-BN, and GT-BC. A multiple alignment of protein sequences was performed using MUSCLE alignment, and a consensus sequence was constructed containing 50 sucrose synthase genes from *Phaseolus vulgaris*, *Medicago truncatula*, *Arabidopsis thaliana*, *Beta vulgaris*, *Zea mays*, *Triticum aestivum*, *Sorghum bicolor*, *Selaginella moellendorffii*, and *Physcomitrium patens*.

The results have shown that many regions within the four domains of sucrose synthase were conserved in all the genes of the studied species including monocots, dicots, lycophyte and Bryophyte, which means that these residues were conserved throughout the evolution of these genes. For instance, in the sucrose synthase enzyme, the residues PDTGGQ (located in 324–329 of the consensus sequence), VYILDQV (339-345), and DFII (510-512) are in the GT-BC domain. Similarly, in the GT-BN domain, the residues GQYE (528-531), FDPKFNI (551-557), and FGLTV (713-717) are conserved (**Supplementary Figure S1**).

Furthermore, the analysis of the predicted conserved domains of the protein that were collected (from the InterPro. software) has shown that the soybean sucrose synthase genes share the glycosyl transferase 4 domains labeled as GT4_sucrose_synthase (**Supplementary Figure S2**).

### 3.6 Soybean mutant library development

The TbyS library was established with 7296 selected M2 mutant families. Young leaf tissues were collected from M2 plants, from which genomic DNA were extracted, quantified, and normalized. In total, 76 plates containing 7144 DNA samples from different M2 mutant families were pooled into six plates: P1, P2, P3, P4, P5, and P6. The pooling was conducted using the bidimensional-arraying pooling strategy. In 160 horizontal pools (P1 and P2), each pool comprises 48 DNA samples, while 24 DNA samples were contained in 120 vertical pools (P3, P4, P5, and P6) (**Supplementary Figure S3**).

The bidimensional-arraying pooling strategy was used for the high-throughput screening of mutation. This ensures that the DNA sample from each mutant is included twice in this strategy, one time in the vertical and another time in the horizontal pool.

### 3.7 Sucrose synthase mutant retrieval from TbyS

A total of 10 sucrose synthase genes were selected to design probes for amplicon sequencing. The genes are located on nine soybean chromosomes, including chromosomes 2, 3, 9, 13, 14, 15, 16, 17, and 19, with two genes on chromosome 9. Each mutation was identified in one well of the vertical pools and another well of the horizontal pools. The demultiplexing of the vertical and horizontal pools in these two wells was used to determine the mutant that contained this mutation.

### 3.8 Characterization of induced mutations in soybean sucrose synthase genes identified through TbyS

The screening of the induced mutations identified through TbyS in 10 sucrose synthase genes has shown 1095 SNP mutations, including 423 G to A, 454 C to T, and 231 mutations other than the previous two mutation types (**Table 2**). Interestingly, 80% of the discovered SNP mutations are typically EMS-type (G to C to A to T), whereas the other 20% represent the other types of mutations. Within the coding sequences of the sucrose synthase genes analyzed by the TbyS, we observed that the identified mutations led to 713 missense mutations, 53 nonsense, and 329 silent mutations (**Table 2**).

**Table 2:** A summary of mutations in ten sucrose synthase genes identified by TbyS.

Gene id	Amplicon size (bp)	Base changes	Type of base changes			Missense mutations	Nonsense mutations	Silent mutations
			G>	C>	Others			
			A	T				
Glyma.02G240400	5600	114	41	41	32	66	3	32
Glyma.03G216300	6241	117	37	51	29	72	6	39
Glyma.09G073600	6397	122	39	55	28	76	4	42
Glyma.09G167000	5901	95	33	37	25	60	8	27
Glyma.13G114000	5528	82	33	42	7	50	2	30
Glyma.14G209900	5789	114	60	35	19	78	6	30
Glyma.15G151000	10938	109	41	46	22	72	5	32
Glyma.16G217200	5611	119	41	48	30	83	8	28
Glyma.17G045800	5574	139	67	58	14	88	5	46
Glyma.19G212800	6134	97	31	41	25	68	6	23
Total	63713	1108	423	454	231	713	53	329
Percentage of each type of nucleotide change (%)			38.18	41.97	20.85			
Percentage of each type of amino acid change (%)						64.35	4.78	29.69

Based on the number of seeds available at the seed laboratory, a subset of the identified mutants was analyzed for the sugar profiles (sucrose, raffinose, and stachyose content) using the HPLC. The results have shown that the missense mutations (R582W, G249E, A166T, and A260T) identified on the sucrose synthase gene *Glyma.02G240400* in the mutants SL446, F1115, F523, and F203, respectively. These mutants have shown a sucrose content of 9.5%, 9.1%, 6.6%, and 7.6%, respectively, which was increased compared to the wild types used including Forrest and Saluki which showed a sucrose content of 4.8 and 6.1, respectively (**Table 3**).

**Table 3:** A summary of mutants identified by TbyS in the sucrose synthase gene family.

Gene id	Plant id	Amino acid changes	Nucleotides changes	Sucrose	Raffinose	Stachyose
				DW%		
Glyma.02G240400	F1115	G249E	G746A	9.1	0.9	5.2
	SL446	R582W	A1744T	9.5	0.7	4.2
Glyma.09G073600	F523	A166T	G496A	6.6	0.7	4
	F203	A260T	G778A	7.6	0.8	6.7
	F1120	G486*	G1456T	7	0.9	5.6
	SL627	P112L	C335T	7.2	0.8	4.1
	F933	T490I	C1469T	8.4	1	4.6
Glyma.09G167000	F674	P373S	C1117T	7.5	0.8	4.3
	F61	R371K	G1112A	8.3	0.9	4.8
Glyma.14G209900	SL64	G305D	G914A	7.1	0.8	4.9
Glyma.15G151000	F1153	P529S	C1585T	6.9	0.8	4.7
Glyma.17G045800	F1651	P177L	C530T	6.5	0.8	5.2
Glyma.19G212800	F657	G507R	G1519A	6.5	0.8	4
	F903	S30F	C89T	8.5	0.8	4.6
	Forrest WT			4.8	1	4.8
	Saluki WT			6.1	0.9	4.7

Additionally, three missense mutations on the *Glyma.09G073600* sucrose synthase gene including the F203 (A260T), SL627 (P112L), and F933 (T490I) have resulted in an increased sucrose content that reached 7.6%, 7.2%, and 8.4%, respectively. Likewise, a nonsense mutation on the same gene in the F1120 (G486*) mutant has shown an increased sucrose content that reached 7% (**Table 3**). Similarly, two missense mutations on *Glyma.09G167000* sucrose synthase gene including, F61(R371K) and F674 (P373S) have increased the sucrose content that reached 8.3% and 7.5%, respectively (**Table 3**). Furthermore, the two missense mutations on *Glyma.19G212800* sucrose synthase gene including, F657 (G507R) and F903 (S30F) caused a sucrose content increase that reached 6.5% and 8.5%, respectively. Moreover, a missense mutation on each of *Glyma.14G209900* (SL64 (G305D)), *Glyma.15G151000* (F1153 (P529S)), and *Glyma.17G045800* (F1651 (P177L)) resulted in a sucrose content increase compared to the wild type that reached 7.1%, 6.9%, and 6.5% (**Table 3**).

Interestingly, most of the selected mutations that resulted in an increase in the sucrose content, presented in **Table 3**, show either similar or lower raffinose content compared to the wild type.

### 3.9 Genotyping and mutation confirmation

After the mutant was determined through TbyS, seeds from the identified lines were planted in the greenhouse, genomic DNA was extracted, and PCR was performed followed by genotyping using Sanger sequencing to follow the mutation segregation throughout the generations.

The genotyping results of the soybean sucrose synthase genes have shown the following results:

The sucrose synthase mutant SL446 has a mutation on the *Glyma.02G240400* gene, and that showed a sucrose content of 9.5%, was confirmed. The SLM4-2023-446-1–2 confirmed plant is heterozygous. Additionally, the F61 line that has a mutation on *Glyma.09G167000* with a sucrose content of 8.2% was confirmed. The FM3-2023-61–1 plant is homozygous and SLM4-2023-446-1–2 plant is heterozygous (**Table 4**).

**Table 4:** Summary of the soybean sucrose synthase gene genotyping results using sanger sequencing.

Gene ID	Plant ID	Amino acid changes	Nucleotides changes	Plant id	Sucrose	Genotype
Glyma.02G240400	SL446	R582W	A1744T	SLM4-2023-446-1-1	9.50	Homozygous
				SLM4-2023-446-1-2	7.30	Heterozygous
Glyma.09G167000	F61	R371K	G1112A	FM3-2023-61-1	7.46	Homozygous
				FM3-2023-61-2	6.93	Heterozygous
Glyma.09G073600	F1120	G486	G1456T	FM3-2023-1120-1	6.96	Homozygous
				FM3-2023-1120-2	6.67	Homozygous
				FM3-2023-1120-3	7.69	Homozygous
				FM3-2023-1120-4	7.14	Homozygous
				FM3-2023-1120-5	7.14	Homozygous
Glyma.09G073600	F627	P112L	C335T	SLM3-2023-627-1-2	7.36	Homozygous
				SLM3-2023-627-1-3	6.84	Heterozygous
				SLM3-2023-627-1-4	6.60	Heterozygous
				SLM3-2023-627-1-6	7.43	Heterozygous
				Forrest WT	4.8	
				Saluki WT	6.1	

Two mutations on the sucrose synthase gene *Glyma.09G073600* were confirmed. Five plants of the F1120 mutant that has a sucrose content of 7%, including FM3-2023-1120-1, FM3-2023-1120-2, FM3-2023-1120-3, FM3-2023-1120-4, and FM3-2023-1120–5 are homozygous. Additionally, four plants of the F627 mutant line, that has a sucrose content of 7.1%, were confirmed and SLM3-2023-627-1-2, SLM3-2023-627-1-3, SLM3-2023-627-1-4, and SLM3-2023-627-1–6 are heterozygous respectively (**Table 4**).

The genotyping results showed that the variability in the sugar phenotypes is most likely due to the segregation of the mutations (heterozygous, revertant wild type, and homozygous mutants).

### 3.10 Homology modeling of sucrose synthase genes

**Figure 5:**
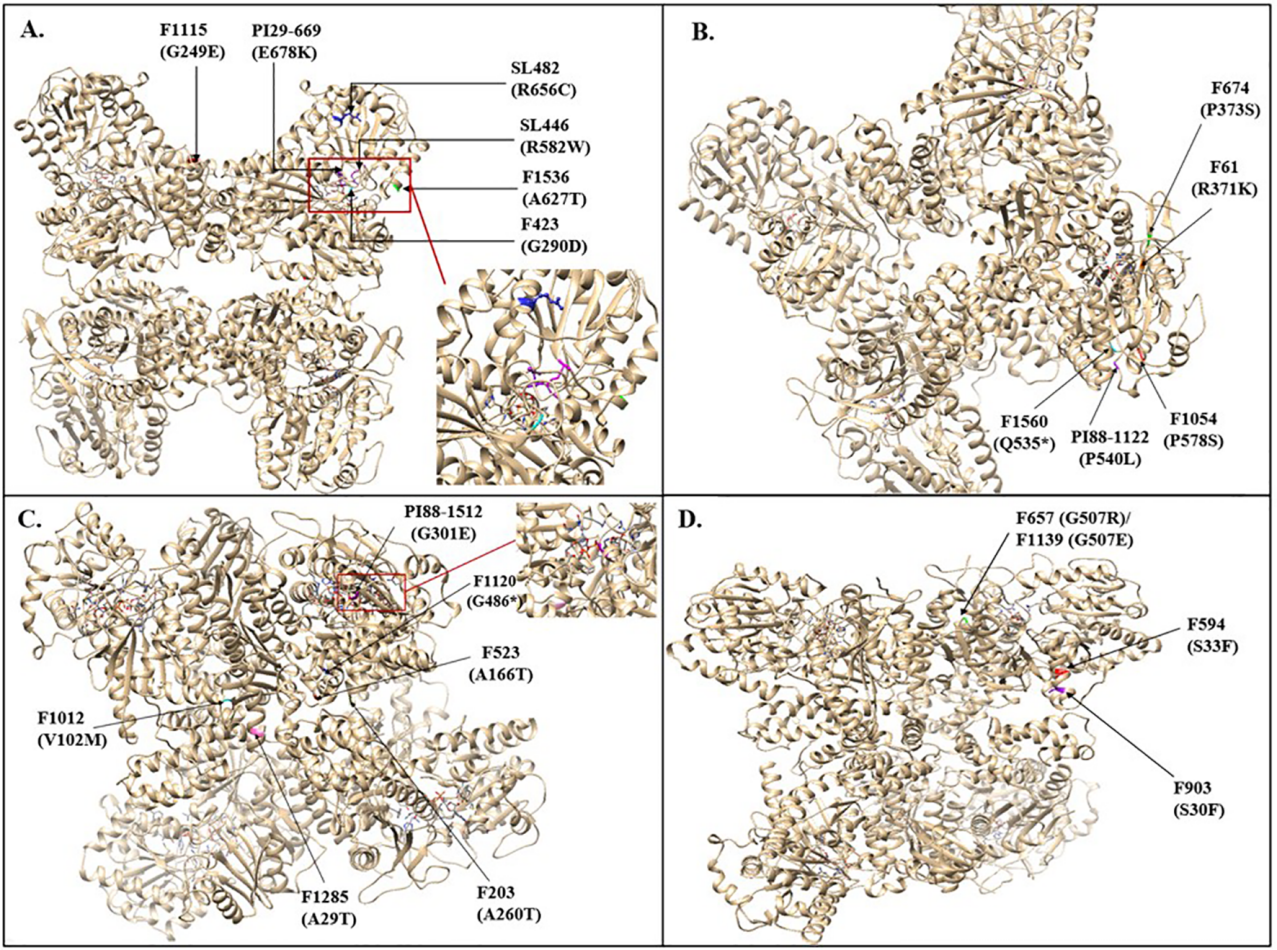
Structural analysis and protein homology modeling of mutants of sucrose synthase genes, including **(A)** *Glyma.02G240400* sucrose synthase gene mutants, **(B)** *Glyma.09G167000* sucrose synthase gene mutants, **(C)** *Glyma.09G073600* sucrose synthase gene mutants, **(D)** *Glyma.19G212800* sucrose synthase gene mutants.

To gain an insight into the impact of the mutations on the protein structure, homology modeling, and structural analyses were performed on four sucrose synthase genes (**Figures 5A–D**). The structural analysis revealed that many EMS mutations were located within the CTD including two selected mutations SL627 (P112L) on the *Glyma.09G073600* gene and F903 (S30F). These two mutants have shown high sucrose content of 8.55 and 7.2%, respectively, compared to the Forrest and Saluki wild types with 4.8%, and 6.1%, respectively.

Additionally, mutations were found in the EPBD domain including F1115 (G249E) on *Glyma.02G240400* gene, F523 (A166T) and F203 (A260T) on *Glyma.09G073600* gene, and F1651 (P177L) on *Glyma.17G045800* gene. These mutants have shown a high sucrose content of 9.1%, 6.6%, 7.6%, and 6.5%, respectively compared to the wild type that has 4.8%. Moreover, mutations were discovered in the GT-BN domain including the selected SL446 (R582W) mutation on *Glyma.02G240400* gene and F1153 (P529S) on *Glyma.15G151000*. This result shows high sucrose content compared to the Forrest and Saluki wild types, which have 4.8%, and 6.1%, respectively. Likewise, many mutations were identified in the GT-BC domain. These include the nonsense mutation F1120 (G486*) on *Glyma.09G073600* along with the missense mutations F933 (T490I) on *Glyma.09G073600*, SL64 (G305D) on *Glyma.14G209900* gene, and F674(P373S) and F61 (R371K), on *Glyma.09G167000* gene, that have shown high sucrose content compared to the wild type reaching 7%, 8.4, 7.1, 7.5, and 8.3, respectively (**Table 3**; **Figure 5B**).

The confirmed mutations were mapped within the sucrose synthase protein (**Figures 5A–D**). The nonsense mutation F1120 (G486*) resulted in a premature stop codon leading to a protein truncation that impacted the formation of the sucrose synthase tetramer which explains the increase in sucrose content (**Figure 5C**).

The SL446 (R582W) mutation on *Glyma.02G240400* gene is located close to the active site which may alter its activity resulting in a high sucrose content. However, the PI29-669 (E678K) mutation was located close to the same active site but it resulted in a decreased sucrose content (2.9% compared to 4.8% of the wild type) which means that it is probably due to increased enzyme activity. Additionally, the F423 (G290D) mutation is located in the same active site loop but it resulted in a sucrose content similar to the wild type (4.8%). This result proves that it does not alter the active site activity. Similarly, the F1512 (G301E) mutation on *Glyma.09G073600* gene is also located in the active site loop of this enzyme. However, the sucrose content of this mutant is lower than that of the wild type (2.2%) meaning that most likely it increases the enzyme activity (**Figures 5A**, **C**). The missense mutation F1115 (G249E) on *Glyma.02G240400* gene was located at the oligomerization site and was therefore predicted to impact the oligomerization of the sucrose synthase protein which explains the increased seed sucrose content in this mutant (**Figure 5A**).

### 3.11 Agronomic performance of the confirmed sucrose synthase mutants

**Figure 6:**
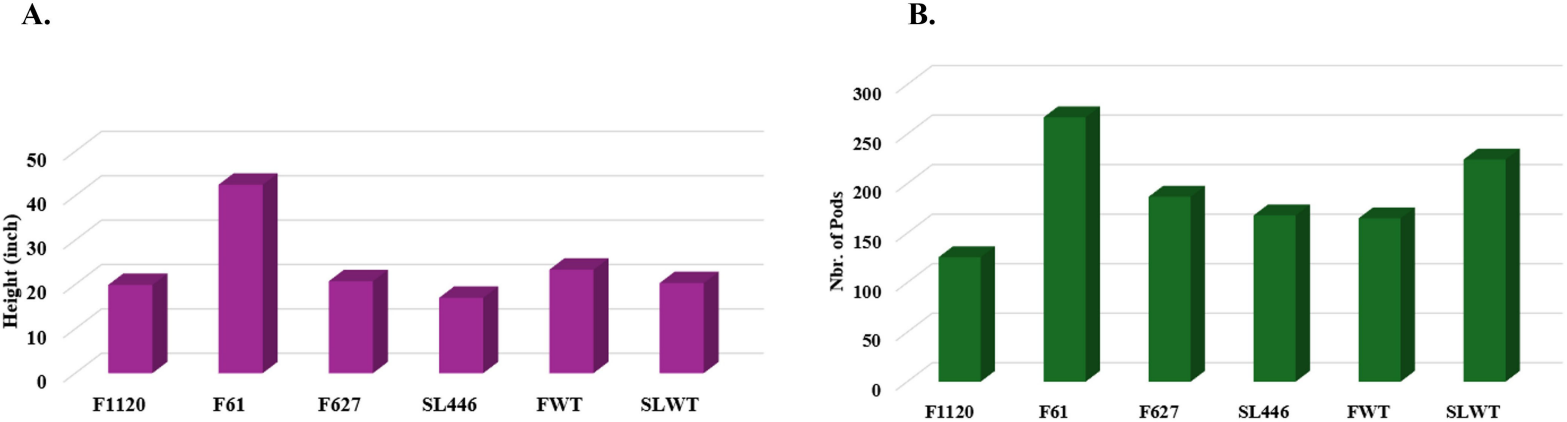
The plant **(A)** height and **(B)** number of pods of the sucrose synthase mutants. One-way ANOVA analysis and Student’s t-test were performed using JMP.

To assess the impact of the confirmed mutations, which resulted in an increased sucrose content on the agronomical performance of the mutant plants, we measured their height and counted the number of seeds they produced in the field. The results show that the height of the plants is not significantly different from the wild type (**Figure 6A**; **Table 5**). Similarly, most of the mutants showed a number of pods that was not significantly different from the wild type (**Figure 6B**; **Table 5**). Furthermore, the pictures have shown that these mutants are performing well in the field and have healthy leaves, branches, and pods suggesting that the confirmed mutations resulting in a sucrose content increase have not altered the plants’ agronomic performance (**Supplementary Figure S4**).

**Table 5:** The number of pods and the plant’s height (inch) of the confirmed mutants.

Gene id	Amino-acid changes	Nucleotides changes	Mutant line	Plant id	Genotype	Number of pods	Height (inch)
Glyma.02G240400	R582W	A1744T	SL446	SLM4-2023-446-1-2	Heterozygous	168	17
Glyma.09G167000	R371K	G1112A	F61	FM3-2023-61-1	Homozygous	195	30
				FM3-2023-61-2	Heterozygous	144	25
Glyma.09G073600	G486*	G1456T	F1120	FM3-2023-1120-1	Homozygous	112	17.8
				FM3-2023-1120-2	Homozygous	176	23
				FM3-2023-1120-3	Homozygous	98	20.9
				FM3-2023-1120-4	Homozygous	138	21.5
				FM3-2023-1120-5	Homozygous	105	16.4
Glyma.09G073600	P112L	C335T	F627	SLM3-2023-627-1-2	Homozygous	111	19
				SLM3-2023-627-1-3	Heterozygous	310	26
				SLM3-2023-627-1-4	Heterozygous	164	18.5
				SLM3-2023-627-1-6	Heterozygous	162	19.5
				FWT		165	23.34
				SLWT		224.4	20.32

## 4 Discussion

The current study characterized the soybean sucrose synthase gene members that were identified earlier (Knizia et al., 2023) through phylogenetic, syntenic, in silico analysis, and TILLING-by-Sequencing+ to identify mutants within these genes.

Sucrose synthase enzyme plays an important role in sugar metabolism, particularly in sink tissues where it facilitates carbon allocation and storage. In most plant species, the sucrose synthase gene family consists of four to seven members (Stein and Granot, 2019). However, soybean has twelve sucrose synthase genes, which is double the number found in other species such as Arabidopsis (Baud et al., 2004), rice (Hirose et al., 2008), *Nicotiana sylvestris* (Wang et al., 2015), and tomato (Goren et al., 2017).

In this study, we conducted an overall phylogenetic analysis of the plant sucrose synthase gene family from nine plant species using the Maximum Likelihood (ML) method. The sucrose synthase genes are classified into three groups I, II, and III, aligning with the previous studies (Xu et al., 2019; Zhu et al., 2017). Group I contains *Glyma.13G114000*, *Glyma.17G045800*, *Glyma.09G073600* and *Glyma.15G182600*. Interestingly, Phaseolus vulgaris genes cluster with almost every soybean gene, most likely due to a common ancestor, especially since they are both leguminous (**Figure 1**). Remarkably, group I is the only group that has a clear separation between monocots and dicots, while group II and group III do not have a clear separation (**Figure 1**). This result is aligned with previous studies (Stein and Granot, 2019; Wang et al., 2015; Zhang et al., 2015; Zhu et al., 2017). This distinctive phylogenetic tree prompts essential questions about the evolutionary history of plant sucrose synthase genes. Stein and Granot, 2019 study investigated this matter and suggested that this is due to the limited number of genes used in these studies. Therefore, they constructed a phylogenetic tree using 133 sucrose synthase genes from 25 plants and successfully constructed a phylogenetic tree with three groups separated into monocots and dicots (Stein and Granot, 2019).

The distribution of sucrose synthase genes has been shown on nine different chromosomes including 9,15, 2, 3, 11, 13, 14, 17, and 20 (**Figure 4**). Most of the sucrose synthase genes are located towards the chromosome ends, indicating probable inter-chromosomal crossovers. This is because of the high genetic recombination rates. The syntenic analysis conducted in this study has shown that soybean sucrose synthase genes expanded through segmental duplications (**Figure 4A**). It is well established that the soybean genome went through two whole-genome duplication events including one that affected the Fabaceae family and the second one that touched only the glycine species (glycine specific) (Schmutz et al., 2010). These duplications resulted in five segmental duplication of the sucrose synthase genes (**Figure 4A**) including *Glyma.02G240400/Glyma.14G209900*, *Glyma.03G216300/Glyma.19G212800*, *Glyma.09G167000/Glyma.16G217200*, *Glyma.09G073600/Glyma.15G182600*, and *Glyma.13G114000/Glyma.17G045800*. The genes in each duplicated pair exhibit a high similarity percentage (over 87.7%) and have co-evolved in the phylogenetic tree, indicating that they are homologous (**Figure 4B**).

The sucrose synthase gene family belongs to the glycosyltransferase-4 subfamily of glycosyltransferases, and its protein functions as a homotetramer (Schmölzer et al., 2016). It has been reported that the sucrose synthase monomer measures approximately 90 kDa and about 800 amino acids in length (Stein and Granot, 2019). The sucrose synthase monomer comprises the glycosyltransferase domain responsible for the enzyme’s glycosyltransferase activity (Stein and Granot, 2019; Zheng et al., 2011). This aligns accurately with our findings for the twelve sucrose synthase candidate genes that have a molecular weight ranging between 92.24 and 105.38 kDa, a protein sequence of 806 to 922 amino acid, and they share the glycosyl transferase four domains.

Many studies have focused on investigating the role of sucrose synthase and have shown that for the *Zea mays* mutant (the shrunken (sh), the mutation has resulted in a 90% reduction in the sucrose synthase activity, decreased seed weight, and a shrunken-seed phenotype (Werr et al., 1985). Likewise, the pea sucrose synthase mutant (rug4) has resulted in a 91% reduction in its enzymatic activity in its root nodules. Those mutant plants lost their capacity for effective nitrogen fixation, even though the nodules appeared normal (Gordon et al., 1999).

In this study, TbyS was used to identify mutants within the characterized sucrose synthase genes. Many mutants have been identified including the sucrose synthase mutants SL446 (R582W) and F1115 (G249E) on *Glyma.02G240400*, which have high sucrose contents of 9.5% and 9.1%, respectively. Interestingly, these mutations have not altered the agronomic performance of the soybean plant.

The discovered sucrose synthase mutants may serve as a promising new resource for enhancing the sugar profile of seeds in soybean breeding programs.

The identified sucrose synthase mutants may serve as a promising new resource for enhancing the seed sugar profile in soybean breeding programs.

## 5 Conclusions

Soybean quality is determined by the composition of its seeds including protein, fatty acid, and sugar content. The soluble sugar content in soybean comprises mainly sucrose and oligosaccharides. Sucrose is desirable because it offers soybean seeds a good taste and high feeding value. The high content of oligosaccharides, including raffinose and stachyose, is undesirable because they are considered anti-nutrients as they are indigestible by humans and monogastric animals. The improvement of soybean seeds nutritional quality by increasing sucrose content and having an optimal level of oligosaccharides (raffinose and stachyose) is a goal for many breeding programs. The sucrose synthase and the invertase are the enzymes responsible for the catalysis of sucrose. In this study, twelve sucrose synthase gene family members were characterized through a comprehensive analysis of the phylogenetic tree, synteny analysis, gene structure, and conserved domain variations. The TbyS+ technology was used to identify mutants within the characterized genes. Many mutants have been discovered, including the sucrose synthase mutants SL446 (R582W) and F1115 (G249E) on *Glyma.02G240400* which have shown a high sucrose content of 9.5% and 9.1%, respectively, without altering the agronomic performance of the soybean plant. The discovered mutations could be useful as germplasm for breeding programs aiming to increase the soybean sucrose content.

## Data Availability

The original contributions presented in the study are included in the article/**Supplementary Material**, further inquiries can be directed to the corresponding author/s.
