# Supplementary material for: Improving Soybean Seed Sucrose Content using TILLING by Sequencing Analyses of The Soybean Sucrose Synthase Gene Family
Source: Front Plant Sci. 2025 Jun 25;16:1606321. doi: 10.3389/fpls.2025.1606321 (PMC12239747; doi:10.3389/fpls.2025.1606321)
Supplement: Supplementary file 2 [file DataSheet2.pdf]

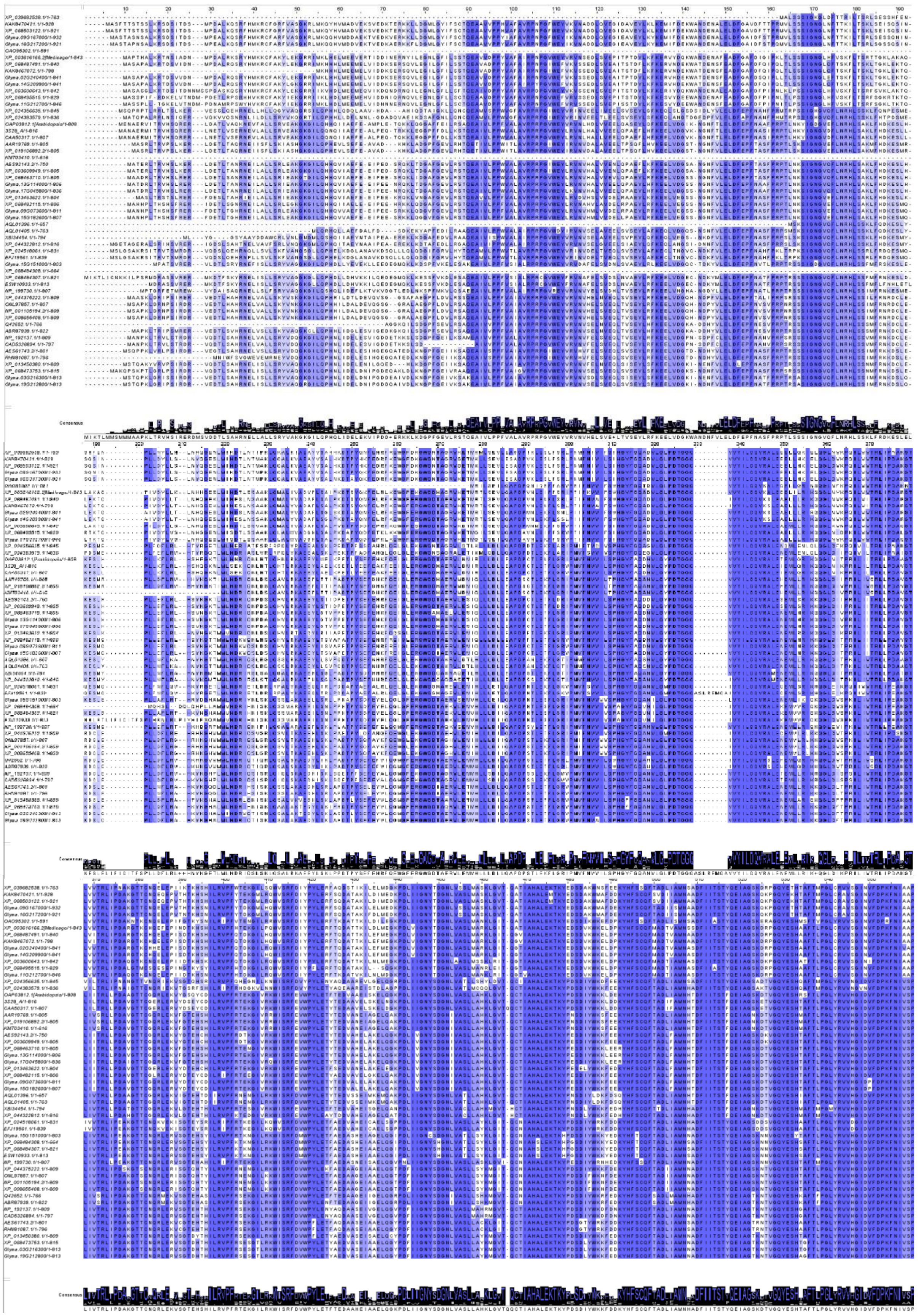
Consensus
Consensus
Consensus
Consensus

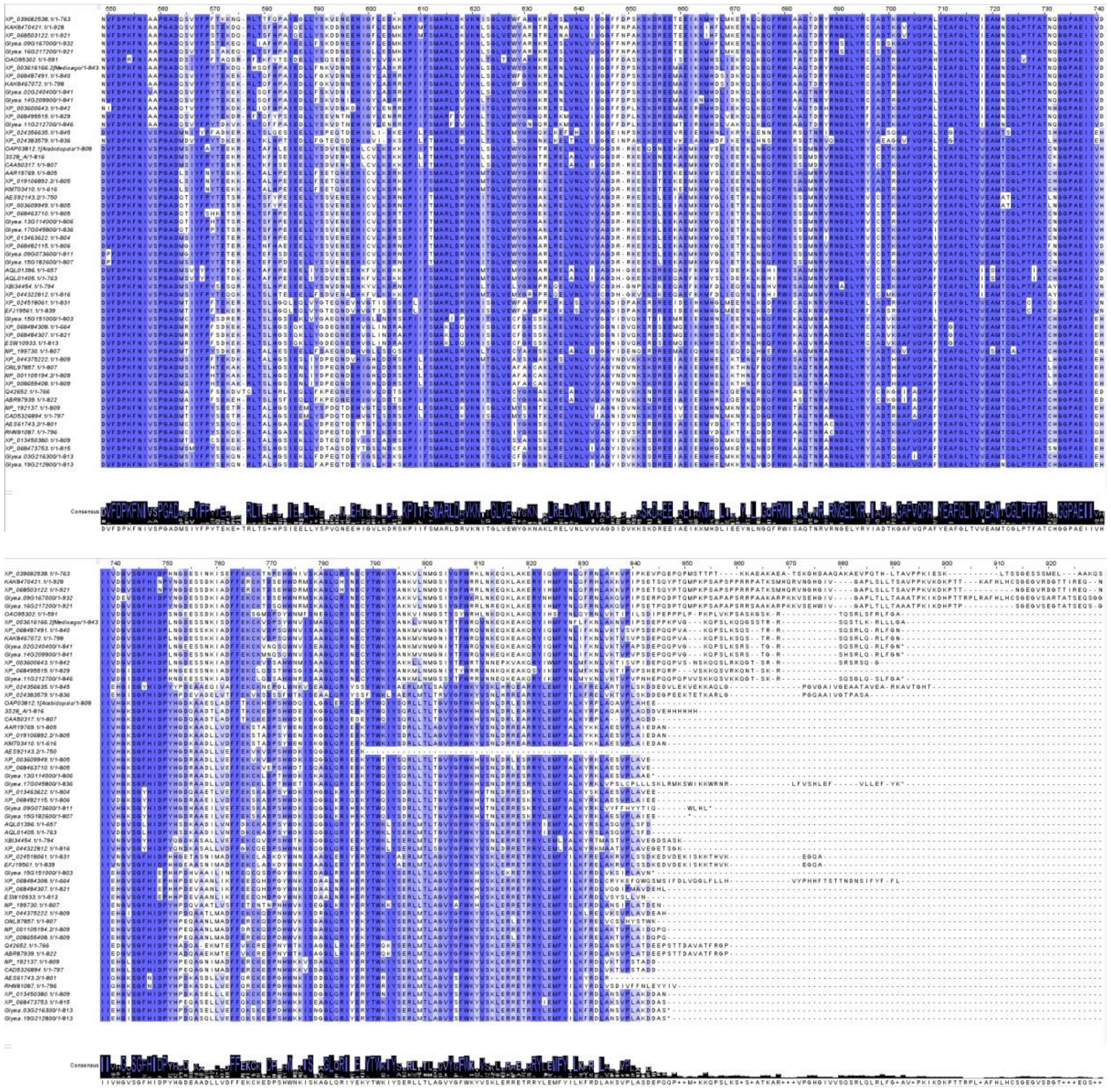

Figure S1. Multiple sequence alignment of sucrose synthase performed using MUSCLE at MEGA 11 and viewed using JalView. The consensus sequence was constructed containing 50 sucrose synthase genes from *Phaseolus vulgaris, Medicago truncatula, Arabidopsis thaliana*, *Beta vulgaris, Zea mays, Triticum aestivum, Sorghum bicolor, Selaginella moellendorffii;* and *Physcomitrium patens.* The color intensity reflects the similarity percentage between the sequences. The darker the color, the highest the similarity percentage.

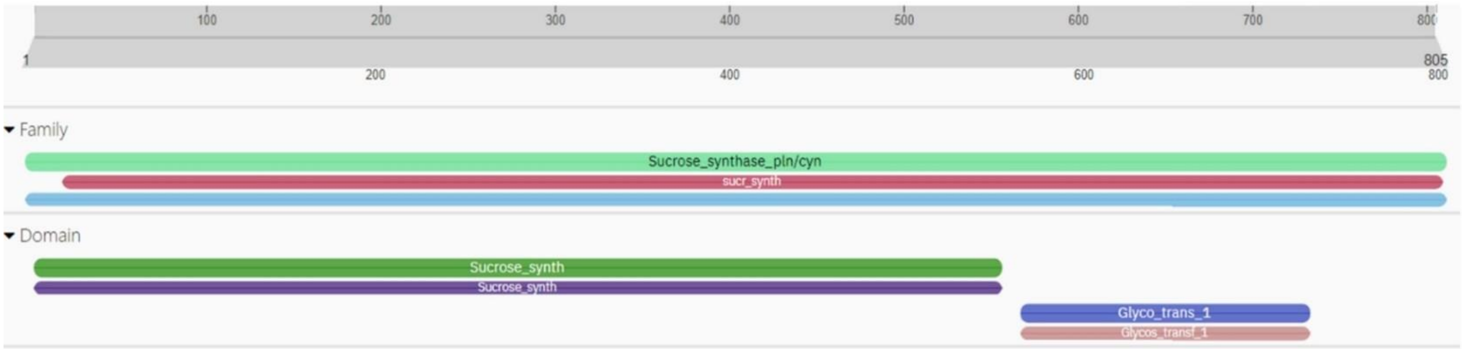

Figure S2. Predicted conserved domains on sucrose synthase gene family. The figure was constructed using InterPro.

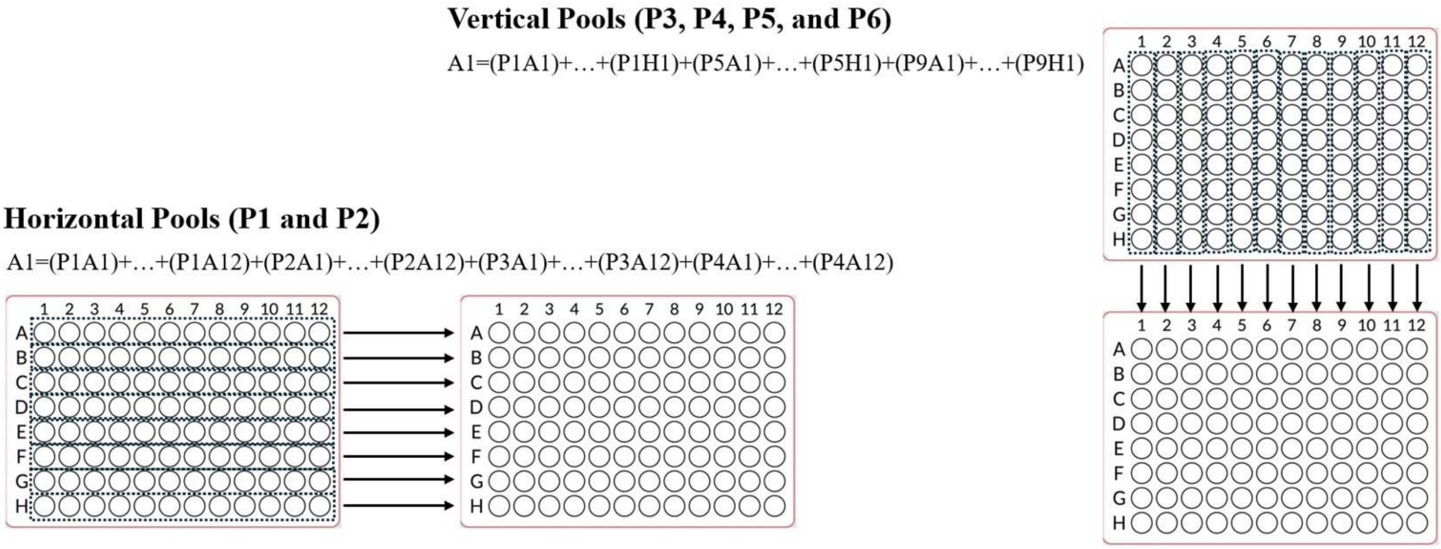

Figure S3. Bidimensional-arraying pooling strategy in soybean mutant library. Using 76 96-well original plates, 160 horizontal pools making two plates called plate 1 and plate 2 (P1 and P2) were formed, with each pool comprising 48 DNA samples, along with 120 vertical pools making four plates called plates 3,4,5, and 6 (P3, P4, P5, and P6) containing 24 DNA samples in each pool.

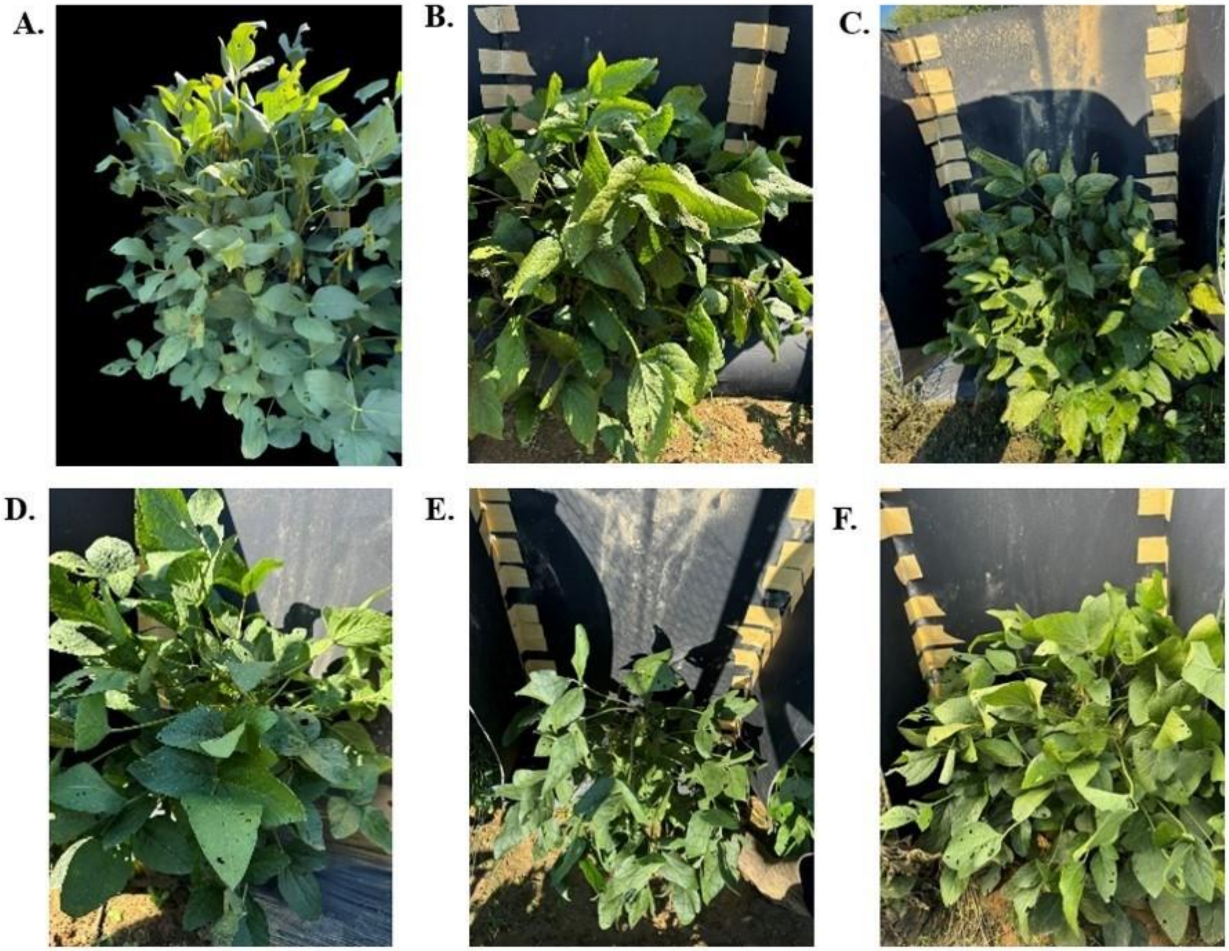

Figure S4. Pictures showing the mutants' performance in the field. (A) F61 (R371K) mutation on *Glyma.09G167000* gene. (B) F1120 (G486*) mutation on *Glyma.09G073600* gene. (C) Forrest wildtype. (D) F627 (P112L) mutation *Glyma.09G073600* gene. (E) SL446 (R582W) mutation on *Glyma.02G240400* gene. (F) Saluki wild type

Table S1. Gene number and accession number of the sucrose synthase gene family.

**Glyma Number**	**Accession Number**
Glyma.02G240400	XM_003518313
Glyma.03G216300	XM_041014172.1
Glyma.09G073600	XM_006587001.3
Glyma.09G167000	XM_003533249
Glyma.13G114000	AC235472
Glyma.14G209900	XM_003544303.5
Glyma.15G151000	XM_026125762.2
Glyma.16G217200	XM_003549139.5
Glyma.17G045800	XM_041010888.1
Glyma.19G212800	XM_041012696.1
